# Multi-locus genome-wide association studies for five yield-related traits in rice

**DOI:** 10.1186/s12870-021-03146-8

**Published:** 2021-08-10

**Authors:** Hua Zhong, Shuai Liu, Tong Sun, Weilong Kong, Xiaoxiao Deng, Zhaohua Peng, Yangsheng Li

**Affiliations:** 1 State Key Laboratory of Hybrid Rice, Key Laboratory for Research and Utilization of Heterosis in Indica Rice, Ministry of Agriculture, College of Life Sciences, Wuhan University, Wuhan, People’s Republic of China 430072; 2 Department of Biochemistry, Molecular Biology, Entomology and Plant Pathology, Mississippi State University, Starkville, MS 39762 USA

**Keywords:** *Oryza sativa*, Yield, ML-GWAS, MLM, QTNs

## Abstract

**Background:**

Improving the overall production of rice with high quality is a major target of breeders. Mining potential yield-related loci have been geared towards developing efficient rice breeding strategies. In this study, one single-locus genome-wide association studies (SL-GWAS) method (MLM) in conjunction with five multi-locus genome-wide association studies (ML-GWAS) approaches (mrMLM, FASTmrMLM, pLARmEB, pKWmEB, and ISIS EM-BLASSO) were conducted in a panel consisting of 529 rice core varieties with 607,201 SNPs.

**Results:**

A total of 152, 106, 12, 111, and 64 SNPs were detected by the MLM model associated with the five yield-related traits, namely grain length (GL), grain width (GW), grain thickness (GT), thousand-grain weight (TGW), and yield per plant (YPP), respectively. Furthermore, 74 significant quantitative trait nucleotides (QTNs) were presented across at least two ML-GWAS methods to be associated with the above five traits successively. Finally, 20 common QTNs were simultaneously discovered by both SL-GWAS and ML-GWAS methods. Based on genome annotation, gene expression analysis, and previous studies, two candidate key genes (*LOC_Os09g02830* and *LOC_Os07g31450*) were characterized to affect GW and TGW, separately.

**Conclusions:**

These outcomes will provide an indication for breeding high-yielding rice varieties in the immediate future.

**Supplementary Information:**

The online version contains supplementary material available at 10.1186/s12870-021-03146-8.

## Background

Rice (*Oryza sativa* L.) is one of the three major food crops supporting more than 50% of the whole population worldwide [1]. In 2018, the total rice output accounted for 32.24% of the total grain production in China followed by maize (http://www.stats.gov.cn/). While in the world, the average production of rice from 1994 to 2019 is 654.78 million tonnes per year, accounting for 27.28% of total cereals output (http://www.fao.org/faostat/en/#data/QC/visualize). The growing global population and the deteriorating environment issue new challenges to the breeding of high-yielding crops [2]. Rice yield is a complex quantitative agronomic trait multiplicatively governed by three major components as the number of grains per panicle, thousand-grain weight, and the number of panicles per plant [3]. Besides, grain size (including grain length, width, and thickness) are also closely related to rice productivity [4]. A previous study reported that rice yield was a representative quantitative trait regulated by several minor genes, and a more efficient tool was needed to develop for exploiting these minor QTLs [5]. Many genes have been reported controlling grain size, grain number, and yield. For example, the *GS3* [6] is a major gene controlling rice grain length (GL). A mutation in the second exon changes a cysteine codon (TGC) to a termination codon (TGA) at the protein level, resulting in a diversity of rice GL. The *GW5* [7] is an IQ calmodulin-binding motif family protein, which regulates rice grain width and weight. Loss-of-function *gw5* showed wider grain compared to the wild type. The *WTG1/ OsOTUB1* [8] encodes an otubain-like protease with deubiquitination activity, which expresses in developing grains and panicles. The overexpression of *WTG1* showed narrow, thin, and long rice grains as a result of slim cells. The *OsSPL13* is a SQUAMOSA promoter-binding-like protein [9, 10], which was reported controlling rice grain length, grain number, grain size, and yield.

The genome-wide association study (GWAS) has become a powerful tool for mining QTL associated with complex traits [11, 12]. Single-locus GWAS (SL-GWAS) methods such as mixed linear model (MLM) [13], efficient mixed-model association eXpedited (EMMAX) [14], and factored spectrally transformed linear mixed models (FaST-LMM) [15] have been widely used to investigate tremendous genetic variants for agronomic traits. However, these SL-GWAS methods are limited in detecting marginal effects QTNs influenced by the polygenic background and stringent Bonferroni correction [16].

To address the shortcomings of SL-GWAS, multi-locus GWAS (ML-GWAS) has been developed as a multi-dimensional genome scan method in which the effects of all markers are estimated at the same time [17]. In particular, to solve the problem associated with co-factor selection in the ML-GWAS model when there are many markers, the mrMLM package was proposed, which containing the following six ML-GWAS methodologies: mrMLM (multi-locus random-SNP-effect MLM) [16], FASTmrMLM (fast mrMLM) [18], ISIS EM-BLASSO (iterative modified-sure independence screening expectation-maximization-Bayesian least absolute shrinkage and selection operator) [19], pKWmEB (integration of Kruskal-Wallis test with empirical Bayes) [20], FASTmrEMMA (fast multi-locus random-SNP-effect efficient mixed model analysis) [21], and pLARmEB (polygenic-background-control-based least angle regression plus empirical Bayes) [22]. ML-GWAS also has a lower false-positive rate and has been applied successfully to identify significant QTNs with subtle contributions for several agronomic [23–26]. But no studies have focused on ML-GWAS for yield-related traits in rice as yet. In general, the QTL (Quantitative Trait Loci) refers to the signal identified by single-locus methods, such as GLM, MLM, *etc*. In such QTL, it mostly contained numerous associated SNPs (Single nucleotide polymorphisms). While in multi-locus GWAS methods, when all the potentially associated markers were identified in the first step. These markers were submitted into a model in further analysis and true QTNs (Quantitative Trait Nucleotides) were further confirmed by the likelihood ratio test [16].

In the current study, a large-scale natural population of 529 rice accessions with five yield-related traits and 607,201 SNPs was conducted by a hybrid method of one SL-GWAS (MLM) algorithm and five ML-GWAS (mrMLM, FASTmrEMMA, pLARmEB, pKWmEB, and ISIS EM-BLASSO) models. We aim to investigate common QTNs via multiple methodologies and then deduce potential candidate genes to accelerate molecular marker-assisted breeding and boost rice production.

## Results

### Phenotypic variation

Five yield-related traits (including GL, GW, GT, TGW, and YPP) were selected to examine whether significant phenotypic variances exist in the yield among the 529 rice varieties. The results manifested that the parameters were varied for accessions to their corresponding traits (Table S1). For instance, the GL ranged from 6.13 to 10.97 mm, with a mean of 8.57 mm. The YPP had a great variation ranging from 4.41 to 92.66 g, whereas the GT possessed the smallest range from 1.61 to 2.45 mm with a CV of 6.86%. The CV of GL, GW, TGW, and YPP were 10.25, 12.36, 16.09, and 44.90%, respectively. Also, the frequency distributions of all five traits obeyed approximately the normal distributions.

**Fig. 1:**
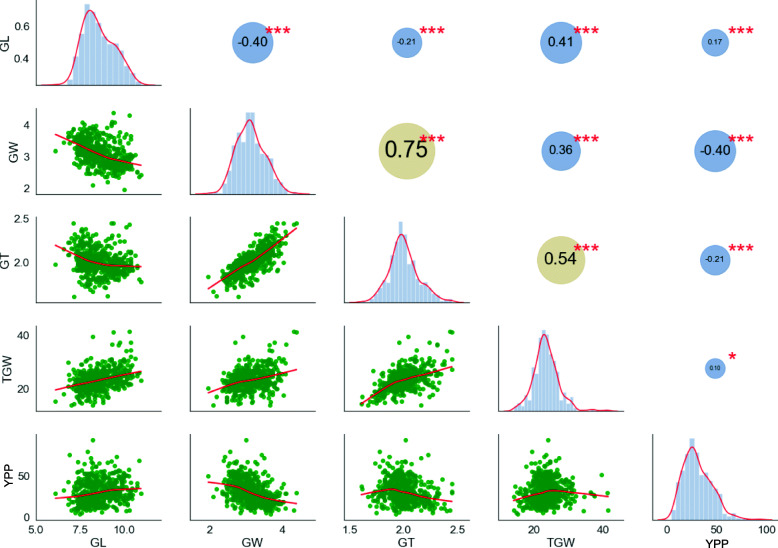
Distribution of five yield-related traits in rice and Pearson coefficient analysis. The lower left represents the linear regression statistics between each two traits, the diagonal histogram represents the distribution of each trait, and the upper right number represents the correlation coefficient (positive numbers represent positive correlation, negative numbers represent negative correlation); asterisks represent significance (* stands for *p*-value less than 0.05; *** stands for *p*-value less than 0.001); yellow circles indicate that the absolute value of the correlation is greater than 0.5, and blue represents less than 0.5

Furthermore, the Pearson correlation coefficients (PCC) among the five traits were also estimated (Fig. 1). All paired traits showed statistically significant differences at the *p*-value< 0.001 except the relationship between TGW and YPP (*p*-value< 0.05). GL and GW had a negative relation with PPC = − 0.40, which was corresponding with a previous study [27]. GL was also associated negatively with GT (PCC = -0.21) while positively with TGW (PCC = 0.41) and YPP (PCC = 0.17), respectively. In addition, TGW was observed to positively correlate to GT (PCC = 0.54), GL (PCC = 0.41), and GW (PCC = 0.36), indicating grain size might make a major contribution to grain weight. These results exhibited that there was a close relationship among the five rice traits, which played an important role in regulating the rice grain shape and productivity.

### Population structure and linkage disequilibrium analysis

**Fig. 2:**
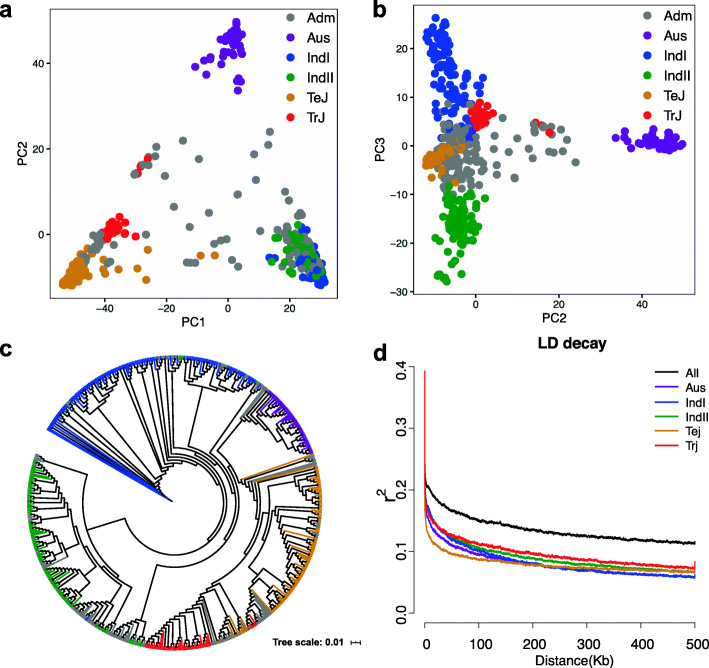
Genetic structure of the 529 rice panel. (**a** - **b**) PCA plots of the 529 rice core varieties. PCA plots present the genetic variation in the rice accessions with PC1 and PC2, PC2 and PC3, separately. (**c**) Phylogenetic tree clustering of 529 rice core germplasm accessions. (**d**) Genome-wide LD decay is estimated from all population and subpopulations. The x-axis represents the physical distance and the y-axis represents the average pairwise correlation coefficient (r^2^) of SNPs. The black, grey, purple, blue, green, orange, and red colors represent All, Adm, Aus, IndI, IndII, Tej, and Trj, successively

To understand the population structure of the panel, PCA analysis was performed using 607,201 SNPs, which was mentioned in the Methods section. Five conceivable subpopulations were respectively distinguished via PC1, PC2, and PC3 (Fig. 2a and b). Next, a maximum likelihood phylogenetic tree was analysed by their genetic distances, which was derived from the SNP differences in these genotypes. The population could be divided into six distinct subgroups, 95 indica I (IndI), 74 indica II (IndII), 43 tropical japonica (TrJ), 93 temperate japonica (TeJ), 46 aus, and 178 admixture of the others (Adm), respectively (Fig. 2c). Based on the results from both the phylogenetic tree and PCA, the panel was separated into six groups.

The LD decay distance was further estimated using the identified SNPs. As delineated in Fig. 2d, the genome-wide LD decay rate of all populations was approximately 43 kb, where the r^2^ dropped to half of the maximum value. Due to that, the theoretical average marker density was one SNP per 9 kb. In fact, the actual value of SNP density in the genome already reached 0.6 (kb/SNP) as the distribution of these SNPs within the whole genome was summarized in Fig. S1. Therefore, we concluded that these markers were sufficiently dense for detecting the associated QTNs.

### SL-GWAS and ML-GWAS analyses

All five yield-related traits were analyzed using one SL-GWAS (MLM) to identify QTL and five ML-GWAS (mrMLM, FASTmrMLM, pLARmEB, pKWmEB, and ISIS EM-BLASSO) methods to identify QTNs (Fig. S2). As for MLM, 152, 106, 12, 111, and 64 SNPs corresponding to 3, 19, 8, 53, and 56 QTLs were found to be tightly associated with GL, GW, GT, TGW, and YPP under the cut-off criterion of *p*-value =1.65 × 10^− 6^, respectively (Table S2). As figured in QQ plots (Fig. S2f and 2i), the curves of GL and TGW were consistent with optimal trends, implying that the false-positive errors were controlled well and the results of the MLM model were reliable.

A total of 74 significant QTNs (LOD ≥ 3) were simultaneously defined to be associated with the above five objective traits by at least two ML-GWAS methods (Table 1). Among these QTNs, 19, 9, 7, 22, and 17 were found to be associated with GL, GW, GT, TGW, and YPP, respectively. A total of 22 correlated QTNs were distinguished for TGW, which were widely located on all 12 chromosomes. For GW, 9 candidate QTNs were distributed on chromosomes 3, 4, 5, 9, 10, and 12. A total of 17 QTN hotspots were detected significantly related to YPP, spread over 2, 3, 4, 5, 6, 7, 8, 10, 11, and 12 chromosomes. Of these, six QTNs were found simultaneously using at least three ML-GWAS methods (*qYPP-3-1*, *qYPP-4-2*, *qYPP-5-2*, *qYPP-7-2*, *qYPP-10-1*, and *qYPP-10-2*). Notably, *qYPP-7-2* was determined across all five ML-GWAS approaches, explaining the 0.28 ~ 1.79% of the phenotypic alteration. Five QTNs (*qTGW-5-1*, *qTGW-5-2*, *qGW-5-2*, *qYPP-7-2*, and *qYPP-10-2*) were mapped by four or more ML-GWAS models.

**Table 1:** The significant QTNs for five rice yield-related traits detected simultaneously by using two or more multi-locus GWAS methods

Trait	QTN	Chr	Position	LOD	R^**2**^ (%)	Method	Reported genes
Grain Length	*qGL-1-1*	1	16,255,794	3.57–6.37	0.29–1.91	1,2,3	
	*qGL-3-1*	3	8,800,352	3.43–4.15	1.71–3.56	4,5	
	*qGL-3-2*	3	16,182,203	3.42–5.81	1.92–2.23	1,3	
	*qGL-3-3*	3	16,699,322	3.68–4.70	2.78–7.54	1,4	*GS3*
	*qGL-3-4*	3	16,717,839	3.47–3.51	2.68–6.97	2,3	*GS3*
	*qGL-3-5*	3	16,911,337	4.01–4.76	3.49–9.75	1,5	*GS3*
	*qGL-3-6*	3	35,509,618	3.22–7.14	2.79–3.68	1,3	*qTGW3*
	*qGL-5-1*	5	5,371,587	4.23–4.92	1.25–2.28	2,5	*GSE5/GW5*
	*qGL-5-2*	5	6,215,765	3.12–5.01	3.91–5.72	1,4	
	*qGL-6-1*	6	4,252,841	4.07–9.18	0.74–1.13	1,3	
	*qGL-6-2*	6	22,641,242	8.54–8.57	7.17–7.35	1,3	
	*qGL-7-1*	7	4,465,180	3.79–11.29	1.01–9.81	1,2,3	
	*qGL-8-1*	8	1,629,499	5.01–5.78	0.67–1.33	1,3	
	*qGL-8-2*	8	14,139,411	3.87–4.67	2.57–3.29	1,3,5	
	*qGL-10-1*	10	4,663,209	4.49–6.46	7.66–13.25	4,5	
	*qGL-10-2*	10	5,288,007	4.05–5.38	1.93–2.63	1,2	
	*qGL-12–1*	12	1,135,876	3.37–3.42	0.00–1.19	2,3	
	*qGL-12-2*	12	5,542,726	4.02–8.98	0.77–1.25	2,3	
	*qGL-12-3*	12	14,546,343	5.74–7.38	0.08–1.73	1,3	
Grain Width	*qGW-3-1*	3	10,817,310	3.50–4.81	0.34–3.64	2,3	
	*qGW-3-2*	3	21,604,259	3.02–3.65	1.62–5.28	1,4	
	*qGW-4-1*	4	26,662,080	3.95–5.95	1.77–2.83	3,4	*STRK1*
	*qGW-5-1*	5	5,359,498	4.12–6.93	1.68–6.34	2,4,5	*GSE5/GW5*
	*qGW-5-2*	5	5,371,587	4.92–11.55	2.83–7.20	1,3,4,5	*GSE5/GW5*
	*qGW-9-1*	9	191,910	3.59–5.35	2.30–2.68	3,4	
	*qGW-9-2*	9	1,318,664	6.74–7.74	5.97–13.97	3,4	*BC12*
	*qGW-10-1*	10	22,500,927	3.31–4.41	1.04–1.50	2,4	*OsCAO1*
	*qGW-12–1*	12	22,677,925	3.04–12.34	0.29–1.47	2,3	
Grain Thickness	*qGT-5-1*	5	4,830,996	12.94–13.56	4.93–7.23	1,4	
	*qGT-5-2*	5	7,022,361	4.94–5.14	1.85–3.20	2,4	
	*qGT-5-3*	5	7,036,290	4.34–13.62	3.32–3.38	1,5	
	*qGT-5-4*	5	23,605,308	3.03–4.41	0.85–5.69	1,3	*OsSNAT1*
	*qGT-6-1*	6	15,688,533	3.43–4.25	3.59–4.17	1,4	
	*qGT-6-2*	6	19,652,114	3.52–10.41	2.23–11.95	1,3	*OsSPDS2*
	*qGT-12–1*	12	17,685,177	3.02–5.90	2.09–3.77	1,4	
1000-Grain Weight	*qTGW-1-1*	1	4,853,002	6.27–6.60	1.97–2.11	1,4	*GW5L*
	*qTGW-1-2*	1	33,427,348	3.19–4.48	0.60–1.57	3,4	
	*qTGW-2-1*	2	2,529,182	4.94–10.21	0.56–5.62	2,3	
	*qTGW-3-1*	3	16,776,481	6.15–7.49	0.89–2.76	1,3,4	*GS3*
	*qTGW-4-1*	4	31,950,052	3.14–4.28	0.42–1.42	3,4	
	*qTGW-4-2*	4	32,409,784	4.54–5.22	1.47–2.41	2,4	*FLO2*
	*qTGW-5-1*	5	4,859,527	7.33–8.91	0.86–3.90	1,3,4,5	
	*qTGW-5-2*	5	7,115,594	4.35–12.71	0.34–4.51	1,3,4,5	
	*qTGW-7-1*	7	18,639,992	3.57–11.64	1.39–6.37	3,4,5	
	*qTGW-7-2*	7	18,895,502	4.22–7.48	1.70–4.99	3,5	*OsSPL13*
	*qTGW-7-3*	7	19,391,625	4.26–11.43	0.44–4.99	1,3,5	*OsSPL13*
	*qTGW-7-4*	7	23,476,357	3.77–5.02	0.48–3.88	1,3	
	*qTGW-7-5*	7	26,928,988	3.89–7.12	0.60–1.40	3,4	
	*qTGW-8-1*	8	25,590,176	3.10–4.53	0.34–1.35	3,4,5	
	*qTGW-8-2*	8	26,309,952	5.51–6.94	1.06–1.95	1,3	*GW8/OsSPL16/qGW8*
	*qTGW-9-1*	9	2,009,389	3.01–3.70	0.45–2.50	3,4	
	*qTGW-10-1*	10	2,107,100	5.81–11.33	1.29–5.77	3,4,5	
	*qTGW-11–1*	11	18,100,034	4.54–9.46	0.35–2.83	1,3,4	*OsBDG1*
	*qTGW-11–2*	11	20,067,792	3.52–6.31	0.87–2.95	1,2,4	
	*qTGW-11-3*	11	25,914,037	3.04–7.25	0.88–1.53	3,5	
	*qTGW-12–1*	12	9,394,697	8.15–10.58	1.72–4.77	1,3	
	*qTGW-12-2*	12	16,332,104	3.01–4.30	0.13–0.89	3,4	
Yield Per Plant	*qYPP-2-1*	2	26,731,138	3.01–3.80	1.13–3.13	4,5	
	*qYPP-3-1*	3	7,685,085	4.19–11.64	3.11–5.26	1,4,5	
	*qYPP-4-1*	4	513,812	4.81–5.88	2.67–3.98	1,5	
	*qYPP-4-2*	4	25,868,074	4.96–6.52	2.15–3.25	1,4,5	*LABA1*
	*qYPP-5-1*	5	28,202	3.72–6.29	3.59–4.01	1,5	
	*qYPP-5-2*	5	25,806,082	4.43–8.85	1.17–6.77	3,4,5	*OsRab7*
	*qYPP-5-3*	5	29,791,637	3.71–5.36	2.38–3.89	1,5	
	*qYPP-6-1*	6	15,037,982	5.50–6.92	3.75–3.87	4,5	
	*qYPP-6-2*	6	30,797,769	3.42–4.60	0.65–1.80	2,5	
	*qYPP-7-1*	7	337,826	8.93–9.51	1.86–5.81	1,3	
	*qYPP-7-2*	7	20,669,197	3.08–6.62	0.28–1.79	1,2,3,4,5	
	*qYPP-8-1*	8	79,975	4.58–5.62	1.65–1.86	2,5	
	*qYPP-8-2*	8	19,396,188	3.12–5.64	0.27–3.08	1,3	*PAY1*
	*qYPP-10-1*	10	5,854,315	3.21–4.64	0.33–2.03	2,3,5	
	*qYPP-10-2*	10	20,388,038	3.81–12.61	0.57–6.99	2,3,4,5	
	*qYPP-11–1*	11	994,705	3.40–3.62	0.59–2.97	3,4	*ONAC122*
	*qYPP-12–1*	12	10,578,786	3.15–4.20	1.65–3.60	4,5	

Methods 1–5 represnet mrMLM, FASTmrEMMA, pLARmEB, pKWmEB, and ISIS EM-BLASSO, respectively

R^2^ (%): the proportion of total phenotypic variance explained by each QTN

**Fig. 3:**
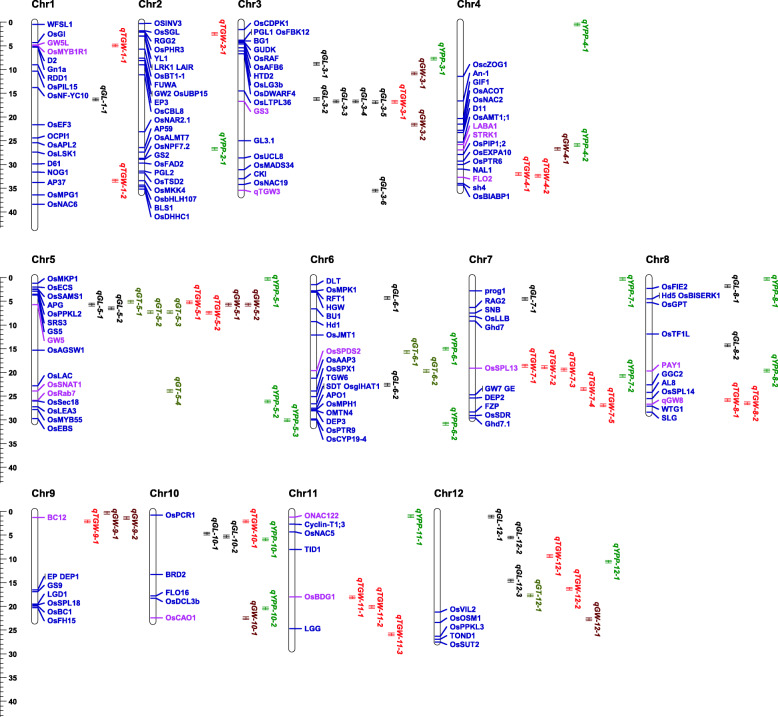
Comparison of location of candidate QTNs and cloned genes on chromosomes. The common QTNs mapped in this study are labeled on the right side of chromosomes, and different colors display different traits: black, GL; red, TGW; green, YPP; brown, GW; olive, GT. Location of known genes is marked on chromosomes, and pink highlights genes covered by QTNs

Moreover, we compared 161 published rice yield-related genes’ locations with 74 significant QTNs and their genomic ranges (300 kb up- and down-stream around the associated QTNs) (Fig. 3 and Table S3). Nearly one-third of QTNs overlapped with the known genes in total. For example, *qGL-3-3*, *qGL-3-4*, and *qGL-3-5* overlapped with *GS3* (*Grain Size 3*). Stunningly, three QTLs, *qGL-5-1*, *qGW-5-1*, and *qGW-5-2*, controlling multiple traits (GL and GW) simultaneously existed in the same region on chromosome 5, which were adjacent to *GW5* (*Grain Width 5*). These regions are generally regarded as pleiotropic. Additionally, we found 51 novel QTLs such as *qYPP-4-1*, *qGL-10-1*, *qTGW-11–2*, and *qTGW-12–1* without coinciding with or adjacent to the known genes.

In addition, we compared the results of ML-GWAS and SL-GWAS and a total of 20 common QTNs were identified (Table S4). Four, four, seven, and five QTNs were discovered associated with GL, GW, TGW, and YPP, respectively. While no common QTN for GT was identified in this study.

### Prediction of potential candidate genes

**Fig. 4:**
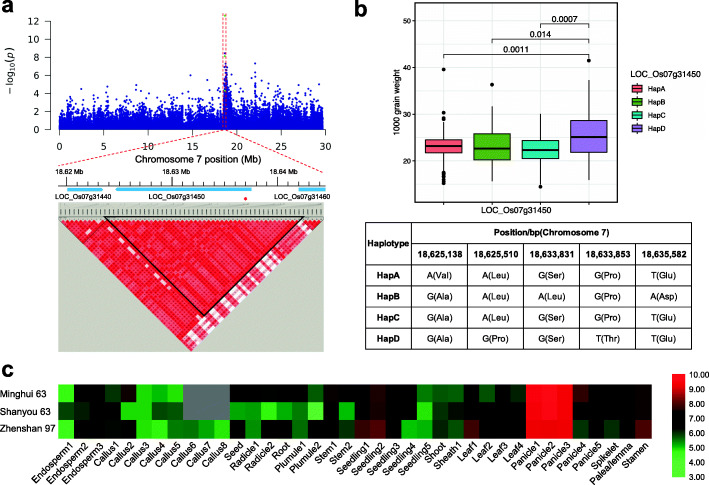
(**a**) Local linkage disequilibrium for *qTGW-7-1.* (**b**) Box plot of TGW traits about four haplotypes of *LOC_Os07g31450*. The x-axis represents four haplotypes of *LOC_Os07g31450* and the y-axis represents 1000-grain weight. The table below is the detailed information of four haplotypes. (**c**) Heatmap of the expression pattern of *LOC_Os07g31450* in various tissues among three local rice species. The y-coordinate indicates three species and relative expression, and x-coordinate indicates 39 different parts and development stages of rice tissue. Red represents higher gene expression and green indicates lower gene expression level, the gene expression levels are log_2_ transformed

Only the QTNs simultaneously detected by both ML-GWAS and SL-GWAS were further analysed. The *qTGW-7-1*, which was located at 18,639,992 bp on chromosome 7, was identified associated with TGW using both SL-GWAS and ML-GWAS methods. This QTN was detected tightly related to TGW with LARmEB, pKWmEB, and ISIS EM-BLASSO methods with the LOD ranged from 3.57 to 11.64 (Table 1). In the MLM method, this SNP was also significantly (*p*-value = 3.62× 10^− 9^) associated with TGW with an R^2^ of 7.79% (Table S2). Then, a local LD block (18,623,910–18,644,333 bp) was defined (Fig. 4a) with the step we mentioned in the Method section. Based on genome-wide annotation information, *LOC_Os07g31440* and *LOC_Os07g31450* were extracted from this region. Among them, *LOC_Os07g31440* encodes an expressed protein with unknown function. The *LOC_Os07g31450*, also known as *CHR729*/*CRL6*, is a CHD (Chromodomain, helicase/ATPase, and DNA-binding domain) protein. Then, we defined four haplotypes of *LOC_Os07g31450* (HapA, HapB, HapC, and HapD) based on the missense mutations in the gene. The accessions with the favorable HapD displayed significantly higher TGW than those with the HapA, HapB, and HapC types (Fig. 4b). These findings revealed that the grain weights of the accessions with favorable haplotype variations were predominantly improved compared to those with unfavourable variations. A previous study reported that *CHR729* expresses ubiquitously, such as in stems, leaves, leaf sheaths, young panicles, and flower organs [28]. To further explore the expression pattern of *LOC_Os07g31450* in different tissues, we utilized the CREP database to analyse and found *LOC_Os07g31450* had the highest expression level in the young panicles (< 1 mm, 3–5 mm, and 10–15 mm) (Fig. 4c).

The *qGW-9-2* was another QTN simultaneously detected by both SL-GWAS and ML-GWAS. This QTN was identified significantly associated with GW using pLARmEB and pKWmEB methods with the LOD value of 6.74 and 7.74, respectively. Moreover, this site was also detected by the MLM method with the *p*-value of × 10^− 8^. A 50.7 kb LD block (1,314,833–1,365,534 bp) was defined for this QTN. In this region, *LOC_Os09g02830* is annotated as *OsMADS78* which belongs to the MADS-box family. *OSMADS78* has been confirmed to be an important regulator of early seed developmental transition and impacts both rice seed size and quality [29]. Nevertheless, the biological function of *OsMADS78* is far from being understood. Here, our study speculated that *OsMADS78* might contribute to rice grain width regulation.

## Discussion

### Comparison of SL-GWAS and ML-GWAS results

The conventional single-locus methods like the general linear model (GLM) and MLM have been widely implemented to identify genetic variants in many cereals [30–32]. However, these models have certain shortcomings as they neglect the overall effects of multiple loci and suffer from the problem of multiple test corrections for critical values. For example, the stringent threshold leads to missing many robust QTLs, particularly small-effect QTLs in MLM [16]. ML-GWAS methodologies therewith have been developed, such as mrMLM, FASTmrMLM, LASSO [33], and FarmCPU (Fixed and random model Circulating Probability Unification) [34]. After comparing the statistical power of ML-GWAS with SL-GWAS methods, several studies demonstrated that multi-locus methods have lower false-positive error and higher statistical power than single-locus ones [17, 35, 36]. According to each ML-GWAS algorithm has its own characteristics and different QTL detection, investigators generally combine the merits of several ML-GWAS methods to mine target QTL for complex agronomic traits [26, 37, 38].

In the present study, we adopted the MLM model and five ML-GWAS methods to analyse five yield-related traits of 529 rice core germplasms. Consequently, 152, 106, 12, 111, and 64 significant SNPs, while 3, 19, 8, 53, and 56 QTLs were detected by MLM underlying GL, GW, GT, TGW, and YPP, respectively (Table S2).

Likewise, 161, 136, 160, 189, and 171 significant QTNs were identified using ML-GWAS methods linked with the above five traits successively. We noted that the number of QTLs mapped by MLM was less than the QTNs identification of five ML-GWAS algorithms, especially of those about GT and YPP. The previous study observed similar findings in GWAS analysis of soybean seed size, suggesting that the recognition results of the five ML-GWAS methods outperformed those of the two SL-GWAS programs. In addition, the QTNs distribution detected by ML-GWAS approaches were more dispersed compared with MLM. For instance, as described in Fig. S2a, the significant loci identified by MLM were concentrated near *GS3* on chromosome 3. Whereas, many loci on other chromosomes failed to meet the threshold, indicating that it was difficult to find new loci from other chromosomes when applied traditional MLM. Afterward, a lot of significant QTNs presented across ML-GWAS models were not only situated on chromosome 3, but also widely distributed on other chromosomes, among which QTNs examined by at least three methods are worthy of further research. These data explicated that the ML-GWAS algorithms are considered more effective, powerful, and robust when applying to investigate the small-effect QTNs for yield-related traits.

### Comparison of QTLs or QTNs detected in our study and previous studies

Over the past decade, numerous rice yield-related genes such as *GS3*, *GW2*, and *GW5* have been identified and their functional roles were deeply elucidated [39, 40]. Among them, *GS3*, the first molecular characterized QTL for grain size, controls grain weight and length, with minor impacts on grain width and thickness [41]. *GW2* (Grain width 2), negatively regulates grain width [42] and *GW2* homologs in common wheat plays a critical role in the genetic control of grain weight and protein content traits [43]. *GW5* influences grain width and weight acting in the brassinosteroid pathway and overexpression of *GSE5/GW5* resulted in narrow grains [44, 45].

In the current study, we characterized 74 QTNs for five yield-related traits that were simultaneously identified using two or more ML-GWAS methods. Compared with the mapped loci of the previous studies, 23 QTNs and its ±300 kb genomic ranges overlapped the known genes. On chromosome 3, the QTN hotspot (*qGL-3-3*, *qGL-3-4*, and *qGL-3-5*) was located near the region of *GS3* for grain length. The QTN cluster on chromosome 5 (*qGW-5-1*, *qGW-5-2*, and *qGL-5-1*) was mapped nearby *GW5*. Moreover, there were 51 novel QTNs excluded in the genomic regions of the past reports. Therefore, these identified makers may be the potential QTNs controlling rice grain and productivity.

### Dissecting two candidate genes of yield-related traits

Using the efficient mixed-model association, two robust QTLs (*qTGW-7-1* and *qGW-9-2*) were validated with major effects associated with yield-related traits using both SL- and ML-GWAS approaches. In the candidate *qTGW-7-1* related to grain weight, *LOC_Os07g31450*/*CHR729* is a kind of CHD protein, which encoded protein contains 2259 amino acids, belonging to the CHD3 subfamily [28]. *CHR729* has been reported to play an essential role in multiple aspects of rice root and seed development. As an example, *CHR729* can control seedling development through the gibberellin pathway [28] and affect crown root formation through the auxin signalling pathway [46]. In this study, GWAS results inferred that *CHR729* might be related to grain weight, and transcriptional expression analysis reflected that *CHR729* was highly expressed in young panicles. The role played by *CHR729* in regulating rice grain and even productivity is worthwhile for further study and confirmation.

In the candidate *qGW-9-2* involved in grain width, gene *LOC_Os09g02830* (*OsMADS78*) belongs to the MADS transcription factor family. MADS family is a large family with conserved MADS-box domains, whose members widely take part in the key regulatory pathways of plant growth and reproduction (including flower formation) [47]. The OsMADS family in rice participates in controlling flowering time, development of root and seed, especially of flower organs [48, 49]. For instance, *OsMADS16/SPW1*, which is homologous to *APETALA3* in *Arabidopsis*, belongs to the Class B in the ABC model of flower organ development, determining the properties of slates and stamens in rice flower organs [50]. Moreover, *OsMADS13* controls ovule identity [51]. *OsMADS26* negatively regulates resistance to rice blast and drought tolerance [52]. And *OsMADS23*, *OsMADS25*, *OsMADS27*, *OsMADS57*, and *OsMADS61* determine root development [48]. Although there are 75 members in the OsMADS family [53], nearly half of the members’ functions are still unknown. In this study, our findings give a clue that *OsMADS78* may be related to grain width in rice. Recently, Paul et al. reported that *OSMADS78* modulates early seed developmental transition and impacts rice grain length, grain width, 1000 grain weight, and grain quality [29]. This conclusion showed the high reliability of our results, corresponding with this published research which verified the biological function of OSMADS78 by over-expression experiment [29].

## Conclusions

In this study, a total of 74 QTN hotspots were simultaneously detected for five yield-related traits by two or more ML-GWAS methods. Among them, *qTGW-7-1* and *qGW-9-2*, closely associated with TGW and GW separately, were presented across both SL-GWAS and ML-GWAS analyses. Besides, two key annotated genes (*LOC_Os07g31450* and *LOC_Os09g02830*) underlying the above two target genomic ranges were mined. In summary, many robust QTLs and two candidate genes were supposed to potentially modulate grain shape and productivity in rice. This research made a beneficial attempt by a combinatory approach of ML-GWAS methods and will facilitate the detection of yield-related QTNs.

## Methods

### Phenotyping data and statistical analyses

The complete phenotypic records of 529 rice core accessions were downloaded from RiceVarMap v2.0 [54], an integrated dataset of rice genomic variations denoted by Huazhong Agricultural University. This set of germplasm contains diverse rice cultivars. Thereinto, the materials were classified into 299 indica (95 indica I, 74 indica II, 13 indica III, and 117 indica intermediate types), 156 japonica (93 temperate japonica, 43 tropical japonica, and 20 japonica intermediate types), 46 aus, 14 aromatic, and 14 intermediate types.

The yield-related agronomic characters include grain length (GL), grain width (GW), grain thickness (GT), thousand-grain weight (TGW), and yield per plant (YPP) were downloaded from RiceVarMap2 website (http://ricevarmap.ncpgr.cn/phenos/). The detailed information, including experimental design, years, replicate, could be found in a study [55]. Meanwhile, the minimum, maximum, mean, standard deviation, range, and coefficient of variation (CV) for each trait were calculated in Table S1. Pearson correlation analysis for phenotypic data was conducted using the SAS 9.4 software (http://www.sas.com/).

### Genotyping data processing

High-quality re-sequencing raw data of 529 germplasms were derived from RiceVarMap (http://ricevarmap.ncpgr.cn/v1/) [54]. The detailed information of data processing and BCF files could be found on the RiceVarMap webpage. Raw single nucleotide variants (SNV) were processed by PLINK 1.9 software with parameter --maf 0.05 --geno 0.05 --snps-only. Then the genotypic data was imputed by Beagle 5.0 and a total of 607,201 SNPs were left for further analysis [56].

### Clustering analysis, population structure, and LD analysis

The matrix of pairwise genetic distance derived from 607,201 SNPs was implemented to construct phylogenetic trees by SNPhylo with the default parameter [57]. Principal component analysis (PCA) and kinship matrix were performed by the Tassel 5.2 program to estimate the population structure [58]. Linkage disequilibrium (LD) between SNPs was estimated as the squared correlation coefficient (r^2^) of alleles, meanwhile the whole population and sub-populations were implemented by software PopLDdecay [59].

### Genome-wide association study

In our case, GWAS was performed in 529 rice varieties with 607,201 high-quality SNPs. A mixed linear model (MLM) was carried out for single-locus method to evaluate the trait-SNP association analysis for agriculture traits using the Tassel software. The first three principal components (PCs) and kinship matrix were used as covariates to correct population structure for decreasing false-positive rate in MLM. The genome-wide significance threshold (*p*-value = 1.65 × 10^− 6^) was calculated by negative log(1/n, n is the number of SNPs).

Five ML-GWAS methods within the mrMLM R package (https://cran.r-project.org/web/packages/mrMLM/index.html) were used to map candidate QTNs, including mrMLM, FASTmrMLM, FASTmrEMMA, pLARmEB, and ISIS EM-BLASSO. All parameters were set at default values, and the critical LOD score was set to 3 for robust QTNs at the last stage. All these five methods used the PCA and kinship matrices in our study. The Manhattan and QQ plots for GWAS were displayed using the R package CMplot (https://github.com/YinLiLin/R-CMplot). QTNs were named as Q + the initial letter of traits name abbreviations + chromosome number + occurrence sequence [60].

### Identification of putative genes

The QTNs identified by at least two different ML-GWAS methods were regarded as the putative candidate loci. Local LD blocks containing at least two SNPs were calculated with all imputed SNP using the PLINK 1.9 software [61]. The local LD blocks of each significant QTN were determined via confidence intervals described by Gabriel [62]. The LD heatmap was visualized using Haploview software [63]. All the genes located in the LD block of QTNs were extracted for further analysis. By comprehensive analysis of gene annotation (MSU 6.1, http://rice.plantbiology.msu.edu/pub/data/Eukaryotic_Projects/o_sativa/annotation_dbs/pseudomolecules/version_6.1/), protein domain function in previous reports, and transcriptome information (data were deposited in the CREP database, Collections of Rice Expression Profiling, http://crep.ncpgr.cn/), the candidate genes for each trait were further mined.

### Phenotypic difference of candidate genes

The haplotypes of the candidate gene were determined based on the missense SNP, then the Wilcox-test was used to test the phenotypic difference among each haplotype. The characters of each trait were visualized with box plots using the R 3.6.1 language [64].

## Supplementary Information

**Additional file 1: Figure S1.** Distribution of SNP markers on Chromosomes. The x-axis represents the number of SNPs in the 1 Mb window, and the y-axis represents 12 chromosomes of rice. Different colors represent different numbers of SNPs.

**Additional file 2: Figure S2.** Manhattan plots of the SL-GWAS and ML-GWAS for yield. (A-E) Manhattan maps representing five traits of GL, GW, GT, TGW, and YPP, respectively; The x-axis displays the chromosome label, and the y-axis displays -log10 (*p*-value). The dotted and solid gray lines show significant associations between SNPs and phenotype value with threshold levels of *p*-value < 1.65 × 10^− 6^ and *p-*value < 8.25 × 10^− 8^, separately. Red, green, blue, purple, yellow, and grey dots represent mrMLM, FASTmrEMMA, pLARmEB, pKWmEB, ISIS EM-BLASSO, and MLM models. (F-J) QQ plots represent MLM analysis of the above five traits.

**Additional file 3: Table S1.** Statistical analysis of five rice yield-related traits.

**Additional file 4: Table S2.** SNPs and QTLs mapping information in MLM analysis.

**Additional file 5: Table S3.** The 161 published genes and their functional information of yield traits in rice.

**Additional file 6: Table S4.** QTN simultaneously detected by both ML-GWAS and MLM methods.

## Data Availability

The genotype and phenotype datasets analysed during the current study are available in the RiceVarMap (http://ricevarmap.ncpgr.cn/). The raw sequence data are available in NCBI with accession number PRJNA171289.

## Abbreviations

Adm: Admixture
CHD: Chromodomain, helicase/ATPase, and DNA-binding domain
CREP: Collections of Rice Expression Profiling
CV: Coefficient of variation
DL: Drooping Leaf
EMMAX: Efficient mixed model association eXpedited
FarmCPU: Fixed and random model Circulating Probability Unification
FaST-LMM: Factored spectrally transformed linear mixed models
FASTmrEMMA: Fast multi-locus random-SNP-effect efficient mixed model analysis
FASTmrMLM: Fast mrMLM
GL: Grain length
GLM: General linear model
GS3: Grain size 3
GT: Grain thickness
GW: Grain width
GW2: Grain width 2
GW5: Grain width 5
GWAS: Genome-wide association study
IndI: Indica I
IndII: Indica II
ISIS EM-BLASSO: Iterative modified-sure independence screening expectation-maximization-Bayesian LASSO
LASSO: Least absolute shrinkage and selection operator
LD: Linkage disequilibrium
LOD: Logarithm of Odds
ML-GWAS: Multi-locus genome-wide association studies
MLM: Mixed linear model
mrMLM: multi-locus random-SNP-effect MLM
PCA: Principal component analysis
PCC: Pearson correlation coefficient
pLARmEB: polygenic-background-control-based least angle regression plus empirical Bayes
pKWmEBb: Integration of Kruskal-Wallis test with empirical Bayes
QTL: Quantitative trait locus
QTLs: Quantitative trait loci
QTN: Quantitative trait nucleotide
R2: The proportion of total phenotypic variance explained by each QTN
r2: The squared correlation coefficient
SL-GWAS: Single-locus genome-wide association studies
SNP: Single-nucleotide polymorphic
SNV: Single nucleotide variant
TeJ: Temperate Japonica
TGW: Thousand-grain weight
TrJ: Tropical Japonica
YPP: Yield per plant
