# Supplementary figures and images for: Multi-locus genome-wide association studies for five yield-related traits in rice
Source: BMC Plant Biol. 2021 Aug 10;21:364. doi: 10.1186/s12870-021-03146-8 (PMC8353822; doi:10.1186/s12870-021-03146-8)

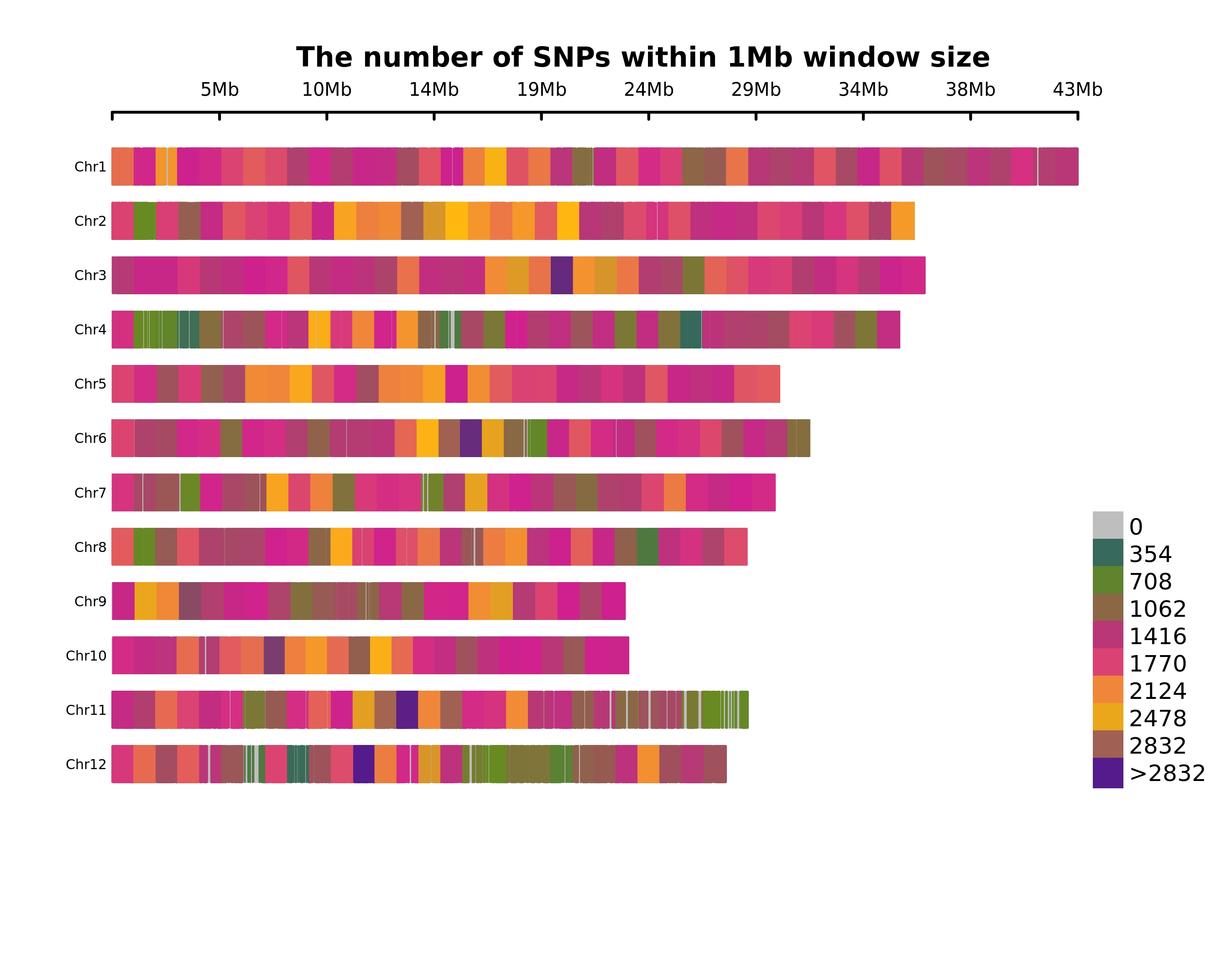

Supplement: Supplementary file 1 — Additional file 1: Figure S1. Distribution of SNP markers on Chromosomes. The x-axis represents the number of SNPs in the 1 Mb window, and the y-axis represents 12 chromosomes of rice. Different colors represent different numbers of SNPs. [file 12870_2021_3146_MOESM1_ESM.jpeg]

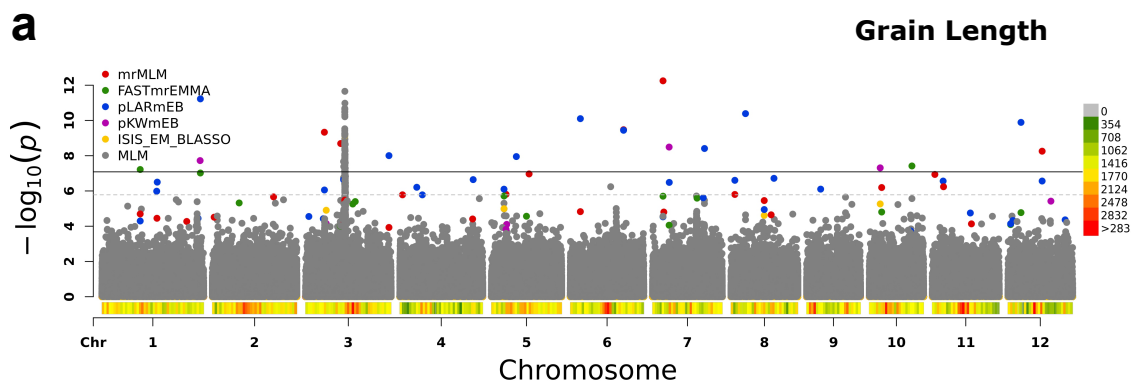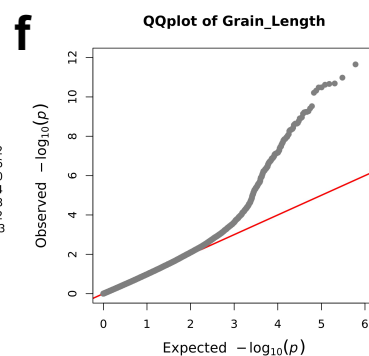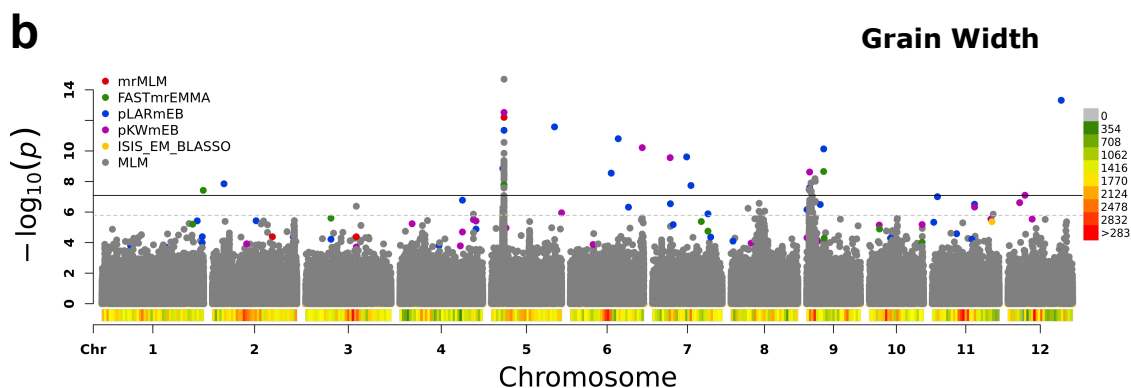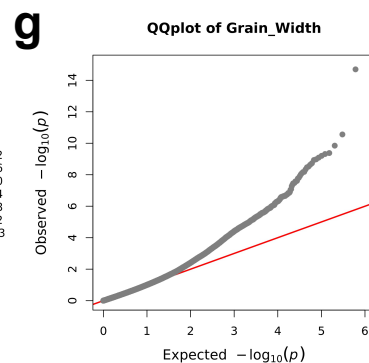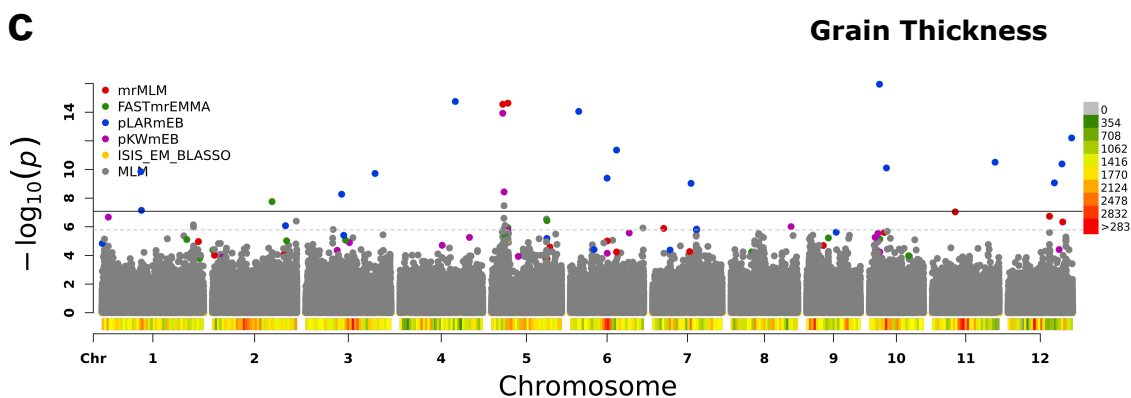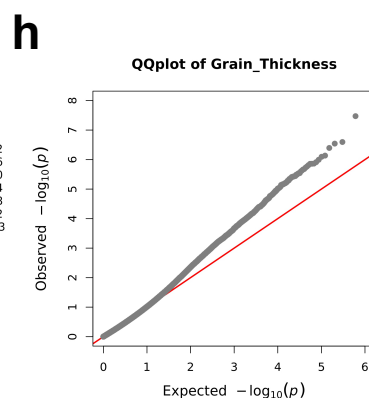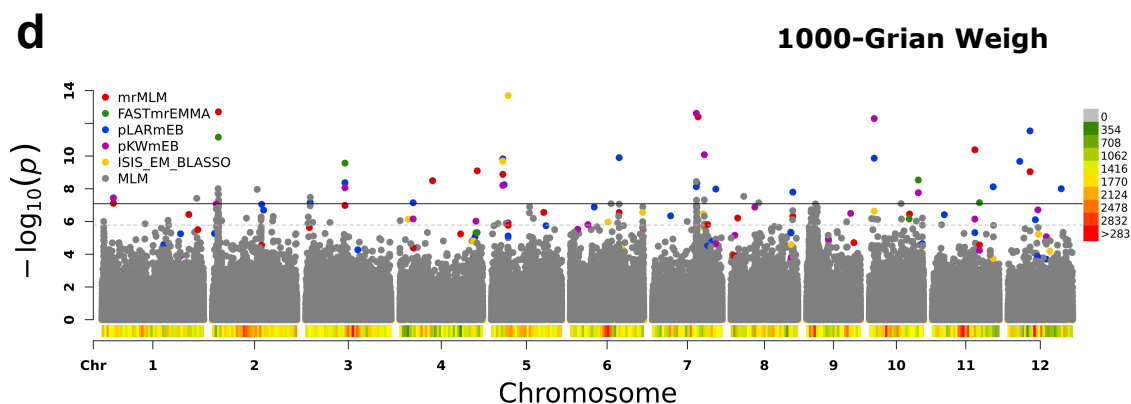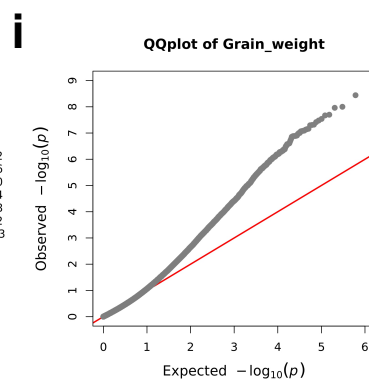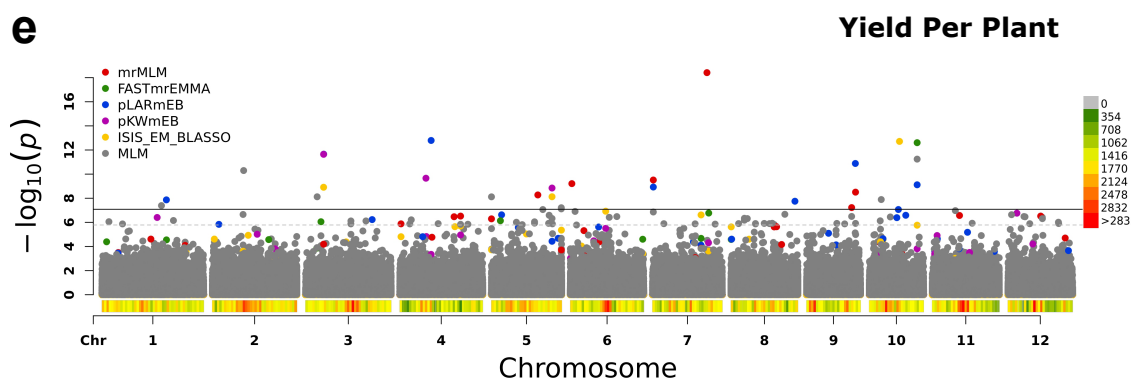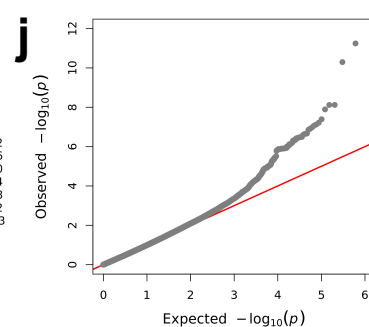

Supplement: Supplementary file 2 — Additional file 2: Figure S2. Manhattan plots of the SL-GWAS and ML-GWAS for yield. (A-E) Manhattan maps representing five traits of GL, GW, GT, TGW, and YPP, respectively; The x-axis displays the chromosome label, and the y-axis displays -log10 (p-value). The dotted and solid gray lines show significant associations between SNPs and phenotype value with threshold levels of p-value < 1.65 × 10− 6 and p-value < 8.25 × 10− 8, separately. Red, green, blue, purple, yellow, and grey dots represent mrMLM, FASTmrEMMA, pLARmEB, pKWmEB, ISIS EM-BLASSO, and MLM models. (F-J) QQ plots represent MLM analysis of the above five traits. [file 12870_2021_3146_MOESM2_ESM.pdf]
